# Quantifying the effect of editor–author relations on manuscript handling times

**DOI:** 10.1007/s11192-017-2309-y

**Published:** 2017-03-03

**Authors:** Emre Sarigöl, David Garcia, Ingo Scholtes, Frank Schweitzer

**Affiliations:** Chair of Systems Design, ETH Zürich, Weinbergstrasse 56/58, CH-8092 Zürich, Switzerland

**Keywords:** Peer review, Collaboration networks, Editorial processes, Social aspects, Empirical studies

## Abstract

In this article we study to what extent the academic peer review process is influenced by social relations between the authors of a manuscript and the editor handling the manuscript. Taking the open access journal PlosOne as a case study, our analysis is based on a data set of more than 100,000 articles published between 2007 and 2015. Using available data on handling editor, submission and acceptance time of manuscripts, we study the question whether co-authorship relations between authors and the handling editor affect the *manuscript handling time*, i.e. the time taken between the submission and acceptance of a manuscript. Our analysis reveals (1) that editors handle papers co-authored by previous collaborators significantly more often than expected at random, and (2) that such prior co-author relations are significantly related to faster manuscript handling. Addressing the question whether these shorter manuscript handling times can be explained by the quality of publications, we study the number of citations and downloads which accepted papers eventually accumulate. Moreover, we consider the influence of additional (social) factors, such as the editor’s experience, the topical similarity between authors and editors, as well as reciprocal citation relations between authors and editors. Our findings show that, even when correcting for other factors like time, experience, and performance, prior co-authorship relations have a large and significant influence on manuscript handling times, speeding up the editorial decision on average by 19 days.

## Introduction

While peer review is undoubtedly a major component in the evaluation of scientific publications, there are different views on the processes that determine which papers are being published, and thus, enter the canon of scientific knowledge. The *reviewer-centric view* considers peer review as a process which is completely driven by *peers*, who decide about the acceptance or rejection of a submitted manuscript. This view acknowledges the role of reviewers, but it typically neglects a second, equally important component in the process, namely the role of the handling editor. In the following, we thus provide a complementary view on the peer review process which emphasizes the important role of the *handling editor* in the fair and unbiased evaluation of scholarly manuscripts.

Prior to the actual peer review process, the editor handling a submission makes a first assessment of (a) its quality, and (b) its suitability for the journal. At a time when more than 50% of submissions to major journals are *desk rejected*, the initial editorial decision to consider a manuscript for further review has become a major hurdle in the publication process. Only after passing this *entry barrier*, the handling editor decides about the reviewers to be contacted. Moreover, a common experience across scientific journals is that many, if not most, reviewer invitations are either not answered at all or declined because of the overwhelming load of review assignments. Thus, finding reviewers that are (1) willing to accept to review a paper, and (2) also deliver their report (in time or after several reminders) has become a considerable challenge. Also here, handling editors play an important role. It is their scientific authority and their close ties to the scientific community which help to choose and acquire competent reviewers. However, this *choice of reviewers* can also possibly *bias the outcome* of the peer review process. Important sources for such a bias are (a) the level of expertise of the chosen reviewer in the topic of the manuscript, (b) potential social relations between reviewers and authors, or (c) the presence of known competing factions in a field that may jeopardize an unbiased evaluation. Developing a (semi-)automated detection of such potential biases that could assist handling editors in the assignment of reviewers and that facilitates a fair and transparent peer review process remains an open challenge. It is thus still the *core competence* of the handling editor to be aware of these issues during the assignment reviewers, or to detect them when assessing the submitted review reports. In summary, it is clear that it is *not* solely the reviewers who decide about acceptance or rejection of a manuscript. The *handling editor* who is able to judge, and to interpret, the review reports and the proposed decisions is—at least—equally important.

In this article we shift the focus from reviewers to handling editors, studying how the latter influence peer review processes. In the following, we summarize some recent works in this area. Given the importance of the handling editor outlined above, it is quite surprising that the current literature about peer review mostly reflects upon the *performance* and *motivation* of editors, rather than on their fundamental role to guarantee a fair and unbiased peer review process.

*Citation benefits for editors* considering potential factors that influence the decision to become an editor, Zsindely et al. (1982) reported a strong correlation between the number of citations that the editors of a journal receive and the impact factor of the journal. This finding by itself does not allow to conclude that editors benefit from the reputation of the journal they edit. However, Lange and Frensch (1999) show that editors, during their assignment as an editor, increase their citation rates in the journal they are editing. Addressing the question whether the citation benefits for editors may influence the outcome of peer review, Levy et al. (2014) investigated to what extent authors preferentially cite the editors of a journal, possibly in an attempt to increase thee chance of their manuscript to be accepted. The results suggest that authors indeed have a tendency to cite those papers which were co-authored by an editor of the journal they are submitting to.

Another line of research has addressed the *performance* of editors, and this performance can be assessed in different (not necessarily orthogonal) dimensions: (1) the ability of editors to attract manuscripts which are later highly cited, which contributes to the journal’s *impact*, (2) increases in publication volume which, in particular for open access journals, affects the journal’s *revenue*, (3) the editors’ ability to handle submissions in a fast, fair and consistent manner, which can improve the journal’s *reputation*, and (4) their contribution to advertise the journal in their scientific community, which increases the journal’s *popularity*. In the following, we highlight works addressing different dimensions of performance of editors.

*Quality control by editors*: Besancenot et al. (2012) study a model which assigns varying levels of *strictness* to editors. They show that the necessary condition for a journal to publish more high quality papers is met when the editors impose a *homogeneous* and *high* level of strictness. However, at the same time Siler et al. (2015) have shown that an overly strict evaluation can result in rejecting high impact papers. While not specifically addressing the role of editors, Bornmann and Daniel (2010) have assessed the performance of quality control mechanisms by measuring the *predictive validity* of editor decisions, depending on the number of peers involved in the review process. This work complements an earlier study by the same authors, which employed a citation analysis of papers initially rejected by a high-impact journal, to assess the amount of *false positives* (type I errors) and *false negatives* (type II errors) in peer review (Bornmann and Daniel 2009). These studies reveal that more careful and unbiased editor behavior often is complemented by higher publication impact.

*Editorial delay of submissions*: Yegros and Amat (2009) introduce the notion of *editorial delay*, defining it as the time difference between the submission and acceptance of a manuscript. They argue that the large variance in editorial delay could indicate potential biases in the review process. Taking into account 13 leading food research journals, they further analyze whether this bias can be traced back to factors such as the country of origin of authors. Their findings suggest that academic experience of authors correlates with shorter editorial delays, while no correlation with the country of origin of authors could be identified. Studying journals in ecology, Pautasso and Schäfer (2010) discovered a statistically significant negative correlation between the editorial delay and the impact factors of the journals in question. Similarly, studying articles from high-profile journals like Nature, Science and Cell, Shen et al. (2015) find a negative correlation between editorial delay and the number of citations of manuscripts. In a subsequent work, some of the authors further advance their methodology, confirming the finding that articles with short editorial delay are more likely to be highly cited (Lin et al. 2016).

*Social biases*: If and to what extent social factors play a role in the scientific discourse, and more specifically the review process, has been studied in a number of works. Crane (1967) studies the validity of the *scientific behaviour hypothesis* which was proposed by Merton (1968). It suggests that personal characteristics of scientists (such as race, religion or gender) influence the way their work is evaluated. Validating this hypothesis, Crane (1967) showed that the knowledge of the academic affiliation of authors has a significant effect on the reviewers’ evaluation of their work. Furthermore, hierarchical structures in science (Cole and Cole 1973) and the social position and prominence of scientists have been shown to play a significant role in how well their works are recognized (Sarigöl et al. 2014; Carayol and Matt 2006). As these complex factors influence the impact of scientific work, similar social phenomena can potentially bias the review process.

Given the general awareness of social biases, it is remarkable that only little attention has been paid to the social relationships between the authors of a submitted manuscript and its handling editor. Garcia et al. (2015) studied the author–editor relationship in a strategic game-theoretical setting. In this paper, we contribute to closing this research gap by means of a large data-driven study of the co-authorship network of authors and editors of a large scientific journal. Specifically, we analyze how a previous collaboration of authors and handling editors in co-authoring a joint paper impacts the handling time of submitted manuscripts. The rest of this paper is organized as follows: The “Materials and methods” section describes the data set as well as our method to construct time-evolving co-authorship networks. Based on the distance between authors and editors in the co-authorship network, in the “Results” section we then analyze the handling times of submitted manuscripts. Apart from this distance, we further discuss several factors that may explain observed reductions in handling time, in particular (1) quality indicators of the submission, (2) reciprocal relations among editors, and (3) topical expertise, connectedness and experience of the handling editor. In the “Discussion and conclusions” section, we discuss our findings and highlight their relevance for the data-driven study (and mitigation) of potential social biases in peer review processes.

## Materials and methods

In this section, we describe (1) the data set used for this empirical study, and (2) our method to construct time-stamped co-authorship networks based on this data set.

### Data set

Our study is based on a large collection of meta-data on more than 100,000 scholarly articles that were published in the multidisciplinary open access journal PlosOne. This data set has been collected based on HTTP requests issued to the web servers of PlosOne with low frequency throughout the month of September 2015.^1^ This approach allowed us to extract meta-data on all articles published between January 2007 and July 2015, namely their titles, abstracts, reference lists, names of authors and handling editors, as well as the dates of their submission and acceptance. Notably, PlosOne makes transparent both the identity of the handling editor of all published manuscripts, as well as the exact times (in daily resolution) when they were submitted and accepted. The data set obtained by the procedure outlined above, provided us with data on a total of 137,536 articles. For 113,335 of those articles, we were able to extract all of the meta-data indicated above. For those 113,335 articles, we additionally applied disambiguation heuristics to uniquely identify editors based on their names and affiliation information. Based on the output of this procedure, we removed 79 publications for which the editor could not be unambiguously identified. As a result, we obtained a data set which comprises 389,960 unique authors and 8110 unique handling editors that we use to construct an empirical *co-authorship network* as explained below. The starting point of our study is to investigate relations between authors and editors of PlosOne in terms of *co-authorships*. Due to the data set used in this work, we can only infer such co-authorship relations based on co-authored articles published in PlosOne, which implies that an editor needs to have published at least one article as author in PlosOne. In our data set, this is the case for 4657 editors and in our study we only focus on those papers that have been handled by those editors.

As a result, this leaves us with a total of 82,742 article meta-data, which serve as the basis for the study of author–editor relations. Each of these published articles $p^i$ can be characterized as a tuple $(t^{i}_{1},t^{i}_{2},{a}^{i},{\hbox {e}}^{i})$, where $t_{1}^{i}$ denotes the submission time of the publication, while $t_{2}^{i}$ represents the acceptance time (both captured with a daily resolution). The sorted list ${a}^{i}=(a_{1}^{i},a_{2}^{i}{\ldots })$ denotes the authors of article $p^i$ while ${\hbox {e}}^{i}$ is the single handling editor. Due to the filtering process mentioned above, each of these handling editors ${\hbox {e}}^i$ is author of at least one other PlosOne publication, i.e. there exists an article $p^j$ such that ${\hbox {e}}^i$ is in the author list $a^j$.

### Constructing the co-authorship network

The procedure outlined above provides us with a time-stamped data set which can be used to construct a *time-evolving co-authorship network* in which *nodes* represent authors of PlosOne publications, while *time-stamped and undirected links* represent co-authored articles published at a given point in time between January 2007 and July 2015. More precisely, we construct time-stamped undirected links (*u*, *v*; *t*) for any pair of authors *u*, *v* that have coauthored an article which was *submitted* at time *t*, i.e. where an article $p^i$ exists such that *u* and *v* are in $a^i$ and $t_1^i=t$. Note that this (common) projection of co-authorship relations for articles with more than two authors implies the construction of fully connected cliques of authors.

**Fig. 1:**
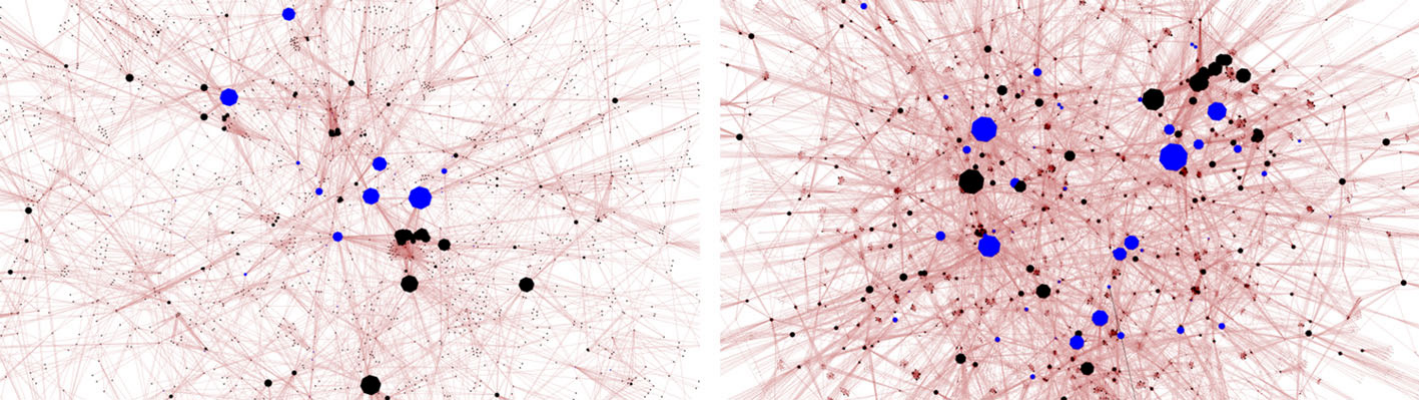
Samples of the largest connected component of the cumulative co-authorship network of PlosOne in July 2009 (*left*) and July 2013 (*right*). *Black nodes* indicate authors of PlosOne, *whereas blue* indicate authors that are also editors of PlosOne. Node sizes are scaled with respect to node degree. *Links* indicate co-authorship relations in PlosOne up to the given time. The networks are magnified to depict the different authors and editors in the two snapshots. (Color figure online)

Finally, our analysis is based on *cumulative networks*, where the cumulative network at time *T* consist of all nodes and links (*v*, *w*; *t*) whose time stamp *t* is smaller or equal than *T*. With the term *cumulative network* we explicitly distinguish our construction from *aggregated* networks, which are often constructed by using all links *irrespective of their time stamps*. In contrast, our construction respects the temporal sequence of link appearances and the cumulative coauthorship network at time *T* only contains those collaborations that have occurred until time *T*. To illustrate our approach, Fig. 1 shows the cumulative co-authorship network at two different times, where authors that are also handling editors are highlighted in blue.

### Performance measures

In addition to data that allow us to reconstruct the *time-evolving co-authorship network*, in our study we also empirically quantify *performance*. In line with the general discussion in the “Introduction” section, the performance of journals and editors can be measured in different ways. We chose (1) the *handling time* of accepted submissions, which impacts the journal’s *reputation*, (2) *the number of citations* of published papers, which reflects the journal’s *impact*, and (3) the *number of downloads* of published papers, which can be seen as a proxy for the journal’s *popularity*.

We quantify the *handling time* $W^{i}=t_{2}^{i}-t_{1}^{i}$ of a manuscript as the time span (measured in *days*) between the date of acceptance $t_{2}^{i}$ of a publication, and the date of its submission $t_{1}^{i}$. The number of *citations* $C^{i}(t)$, and the number of *downloads* $S^{i}(t)$ of a publication $p^i$ are dependent on time *t*, and can only increase or stay constant. For both quantities, we used data available from the web page for each PlosOne publication page at the day of its retrieval in September 2015. In our study including performance measures, we finally consider the number of citations and downloads of articles, however we restrict our analysis to those 48,482 articles that were published between August 2009 and August 2013. As our data set covers PlosOne publications until July 2015, this ensures that for each article we have at least 2 years of citation and download data. This allows us to approximate the scientific impact of an article *i* in terms of the citations $C^i$ and its popularity as the number of downloads $S^i$.

## Results

### Network distance between authors and editors

Based on the time-dependent co-authorship network constructed above, we can calculate the shortest path distance ${\mathtt {dist}}(u,v; t)$ between any pair of nodes *u* and *v* as the minimal number of links that need to be traversed to reach node *v* starting from node *u* in the cumulative network up to time *t*. If *u* and *v* are neighbors, i.e. a direct link between *u* and *v* exists, their shortest path distance is one. If *u* and *v* are not directly connected at time *t* but have one neighbor in common, their shortest path distance is two, etc.

Using this simple network-based distance metric, we define the distance $D^{i}$ between the authors $a^{i}$ of publication $p^i$ and its handling editor ${\hbox {e}}^{i}$ as the minimum of all shortest path distances between the handling editor and any of the authors at submission time $t_{1}^{i}$, i.e.

1 $$\begin{aligned} D^{i} := \min _{a^{i}_{k} \in a^{i}} {\mathtt {dist}}\left( a_{k}^{i},{\hbox {e}}^{i};t^{i}_{1}\right) \end{aligned}$$

As such, a distance $D^{i}=1$ means that the handling editor of publication $p^i$ is a (previous) co-author of at least one of the authors of $p^i$. A distance of $D^{i}=2$ implies that the handling editor and the authors of the publication have at least one common co-author with respect to their publication history in PlosOne, while they have not directly co-authored any article in our data set.

While $D^{i}$ can take any positive, finite value, we define $D^{i}=\infty$ if there is *no* path between the handling editor and any of the authors. This can happen in two cases: (1) the handling editor has not co-authored any paper in PlosOne *prior* to the submission time $t_{1}^{i}$ of article *i*,^2^ or (2) the handling editor has co-authored a paper in PlosOne before $t_{1}^{i}$, however the handling editor and the authors of publication $p^i$ are in different *disconnected components*, i.e. there is no path between authors and handling editor.

In our study, we are particularly interested in the case $D^{i}=1$, i.e. those cases where a co-authorship link between the handling editor and at least one of the authors of the article exists (within the corpus of PlosOne papers). We specifically compare these instances with cases where $D^{i}>1$, i.e. articles where no previous co-authorship relation (within the PlosOne corpus) exists between the handling editor and any of the authors. In our data set, we identified four publications with a network distance of zero, i.e. cases where the handling editor was at the same time one of the authors of the article. Moreover, we identified 1067 publications where the distance is one, while for the remaining 81,671 publications we obtain distances larger than one.

At this point it is important to highlight that our data set only includes information on PlosOne publications, i.e. we necessarily neglect co-authorship relations between authors and handling editors which are due to articles published in other journals. Accounting for such external co-authorship relations turned out to be not feasible for this study, due to the complexities involved to unambiguously match author identities across different bibliographic databases. As such, the co-authorship relations inferred based on our corpus of PlosOne publications provide us with an *upper bound* for the distances $D^i$. While we encourage future replications of our study based on editor–author co-authorship relations across journals, we expect our results to hold since our methodology is likely to underestimate the presence of social relations.

The reader may argue that the number of publications for which the distance between handling editor and authors is one by itself does not allow us to argue about the reasons underlying these editorial assignments. Precisely, even based on a random assignment of handling editors to publications, we can already expect some papers to be assigned to prior co-authors. To study whether the observed number of distance one publications is likely to be based on chance, we compare the empirical network against different randomized versions. For this, we use the final cumulative co-authorship network *G*, emerging at the final time stamp of our analysis. We then consider two random reshuffling models. In the first model, we randomly shuffle handling editors among the publications while preserving all of the co-authorship links of our network *G*. As an additional boundary condition, we shuffle editors in such a way that each handling editor as assigned the same *number* of (possibly different) articles as in the empirical data set. As a result, we obtain a network topology with the same link structure, but where the identities of nodes have been swapped. We denote a random realization *k* of this *randomized editor network* as $G_{k}^{{\mathrm {ed}}}$. For the second model, we maintain all handling editors assigned to each article, while randomly reshuffling all co-authorship links. Here, we additionally respect the boundary condition of preserving the number of co-authorship links of each author, while the targets of those links are randomized. We denote a random realization *k* of this *randomized co-authorship network* as $G_{k}^{{\mathrm {co}}}$.

We can now calculate the minimal distance between handling editor and authors for each publication $p^i$ in realization *k* of the randomized editor network as $D^{i}_{{\mathrm {ed}},k}$. Analogously, we calculate the distance in realization *k* of the randomized co-authorship network as $D^{i}_{{\mathrm {co}},k}$. For each of the two models, we generate 50 random realizations *k*, thus obtaining a distribution of distances based on the union of distance values across all simulations. We finally compare the empirical frequency of distances $N(D_i)$ with the frequency obtained from the randomized editors network $N(D^{i}_{{\mathrm {ed}}})$ and the randomized co-authorship network $N(D^{i}_{{\mathrm {co}}})$, normalizing these two to have the same mass as $N(D^i)$.

**Fig. 2:**
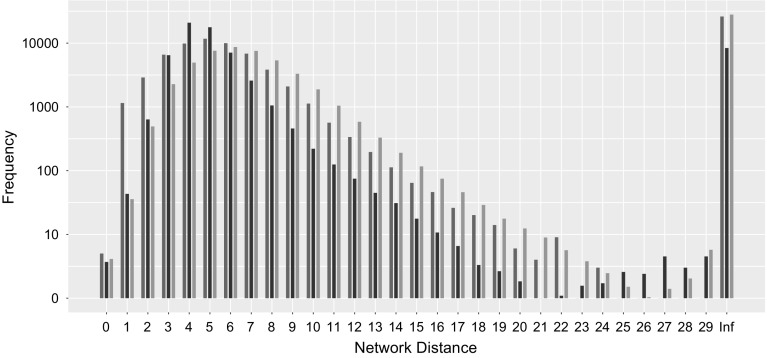
Frequency of publications in the empirical network $N(D^i)$ (*red*) and in the normalized union of 50 instances of the randomized editor network $N(D^{i}_{{\mathrm {ed}}})$ (*blue*) and of the randomized co-author network $N(D^{i}_{{\mathrm {co}}})$ (*green*). (Color figure online)

The results of this analysis are shown in Fig. 2, where the red histogram represents the frequency of distances in the empirical network, while the blue and green histograms indicate the frequency of the randomized editor and co-author network respectively. A comparison of the empirical distribution with those of the randomized models reveals that the number of publications with $D^i=1$ in the empirical data is considerably higher than in both random models. More precisely, in the empirical network the ratio of publications with $D^i=1$ is 27 times larger than in the randomized editor network and 21 times larger than in the randomized co-authorship network (both ratio comparisons pass a $\chi ^2$ test with $p<10^{-15}$). This effect can also be observed at other low distance values, for example the ratio of articles with $D^i=2$ in the empirical network is 5.07 larger than in the randomized editor network and 3.57 times larger than in the randomized co-authorship network. The effects exhibits a similar trend up to $D^i=6$, for which the ratios even out such that the empirical network has a ratio of articles with $D^i=6$ that is 0.99 times the one in the randomized editor network and 1.1 times the one in the randomized co-authorship network.

On the one hand, one could argue that the higher frequency of publications with $D^i=1$ compared to a random model can be explained by the fact that handling editors are more likely to be from the same scientific community as the authors. On the other hand, these findings also indicate that there is seemingly no effective strategy in place to *avoid* cases where editors handle submissions of previous collaborators. The absence of such a strategy can give rise to potential *conflicts of interests* and *social biases* in the handling of manuscript. The list of competing interests for editors in PlosOne includes “published with an author during the past 5 years”.^3^ In our data set, more than 95% of the cases of articles in which the editor previously published with an author happen within 5 years of the previous publication, showing that this competing interest is not effectively prevented. In the following, we thus study the question whether we can identify traces in the data that could result from such biases.

### Effect of network distance on handling times

Unfortunately, using the publicly available data introduced above, we *cannot* investigate whether handling editors are more likely to accept submissions from previous co-authors than from other authors. This is due to the fact that we do not have data on rejected manuscripts. However, we *can* investigate whether handling editors accept submissions from previous co-authors faster than those from other authors. In this section, we study this question by comparing the distribution of manuscript handling times for publications $p^i$ with distance $D^i=1$, to those of publications *j* with $D^j>1$.

As we consider the distribution of manuscript handling times $W^i$ and distances $D^i$ for all publications $p^i$, in the remainder of our article we drop the superscript *i*. Precisely, we denote as *D* the *random variable* representing editor–author distances, and as *W* the random variable representing handling times for a publication. With *P*(*W*) we further denote the (rather broad) *distribution* of handling times. With this, we can address our research question by calculating the conditional distribution $P(W|D=1)$ of handling times of articles with distance one and $P(W|D>1)$ for articles with distances larger than one. Our hypothesis is that $P(W|D=1)$ and $P(W|D>1)$ differ such that the average conditional handling time is smaller for $D=1$ compared to $D>1$. If we cannot find a significant difference between these distributions (or if we find the opposite relation) our hypothesis must be rejected.

**Fig. 3:**
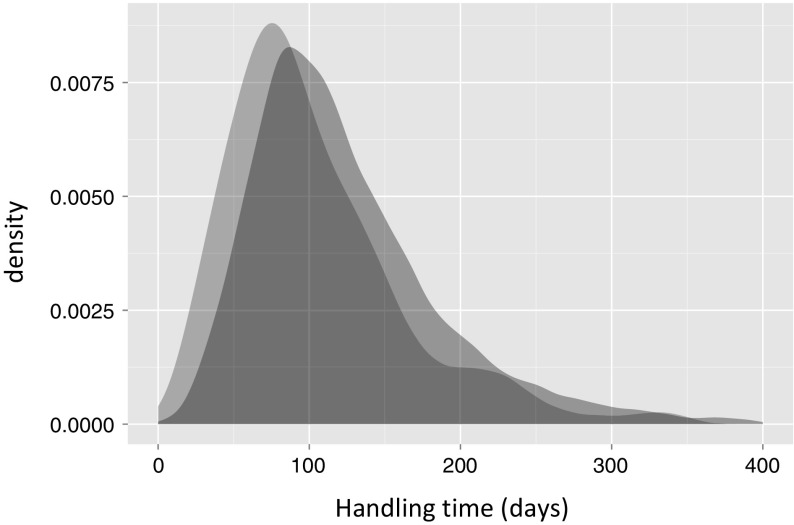
Kernel density plots $({\hbox {bandwidth}} =0.8)$ of the conditional distributions of *W* given $D=1$ (*red*) and $D>1$ (*blue*). There is a significant shift in medians of 19 days. (Color figure online)

Figure 3 shows the two conditional distributions of manuscript handling times for $D=1$ (*red*) and $D>1$ (*blue*). Here, in line with our hypothesis, we visually notice that the distribution $P(W|D=1)$ is considerably shifted toward lower values compared to $P(W|D>1)$. We can confirm this visual observation by means of a *Wilcoxon rank sum test*. This test yields a significant difference of 19 days between the medians of both distributions ($p < 10^{-6}$). In other words, submissions of previous co-authors of the handling editor are, on average, accepted almost 3 weeks faster than submissions of other authors. A similar effect can be observed at $D=2$ in the “Appendix” section Fig. 5, weakening for network distances 3 and longer.

We can alternatively test this speed up in the manuscript handling time through a regression model. To differentiate between $D=1$ and $D>1$, we define an indicator function $\delta _{D,1}$ that takes the value $\delta _{D,1}=1$ if $D=1$ and $\delta _{D,1}=0$ otherwise ($D>1$). With this, we can model the dependence of the handling time on $\delta _{D,1}$ as a *log-linear regression model* in which the dependent term $\log (W)$ is expressed as

2 $$\begin{aligned} \log (W) = \alpha + \delta _{D,1} \alpha _{1}. \end{aligned}$$

Equation (2) corresponds to a model in which the average handling time is a constant, that differs dependent on the author–editor distance *D*. The intercept $\alpha$ captures the baseline handling time for *all* publications, whereas $\alpha _{1}$ measures the *additional* effect on the handling time for publications in which the handling editor is a previous co-author of at least one of the submitting authors. We refer to this as Model 1 and the results of the model fitting are given in Table 1. The significantly negative estimate of $\alpha _{1}$ confirms the reduction of the handling time for articles where handling editors have prior co-authorship relations to one of the authors.

**Table 1:** Regression results for models including performance metrics

	Model 1	Model 2	Model 3
$\alpha _1$	−0.172***	−0.166***	−0.205***
$\beta ^c$			−0.116***
$\beta ^c_{1}$			0.023
$\beta ^s$		−0.120***	
$\alpha$	4.664***	5.401***	4.882***
*N*	48,482	48,482	48,482
Log lik.	−44,283	−43,718	−43,455
AIC	88,571	87,442	86,918

* $$p < 0.05$$; ** $$p < 0.01$$; *** $$p < 0.001$$

We finally test the significance of our findings by comparing them to the randomized editor and co-authorship networks described in “Network distance between authors and editors” section. For this, we first generate 50 random realizations according to the two random models. For each of these realizations, we then calculate editor–author distances and calculate the distribution of handling times for $D=1$ and $D>1$ articles separately. Analogous to Fig. 3, the resulting distributions are shown in Fig. 6 in the “Appendix” section. Different from the empirical distributions, here we cannot identify a clear difference. Moreover, a Wilcoxon test confirms that for both models there is no significant shift between the handling time distributions (obtaining $p = 0.99$ and $p=0.23$ for randomized editors and randomized coauthors respectively).

### Accounting for the effect of performance on handling time

It is tempting to attribute the fact that submissions from previous co-authors are accepted significantly faster to social biases or favoritism. However, a simple alternative explanation could be that these publications are accepted *faster* because they are, in some objective sense, *better*. A reason for this could be that handling editors, who are likely to be reputed and experienced scholars, are likely to have co-authored articles with other reputed scientists. As such, the conjectured bias could, in fact, be a quality bias rather than a social bias that is due to social relations between authors and editor (Table 1).

To account for this effect, we should thus test whether those articles that are handled faster are also of higher *quality*. While it is impossible to objectively and automatically assess the quality of a research article, in what follows we proxy quality by means of the simple performance metrics introduced in the “Performance measures” section. We particularly use the number of citations $C^{i}$, and the number of downloads $S^{i}$ of a publication $p^i$.

Like above, in the following we are interested in the distribution of these quantities. We thus again drop the superscript and introduce the random variables *C* and *S* respectively. We can now test for possible correlations between the variables *C* and *S* and manuscript handling times by means of a correlation analysis. Since the distribution of citations and downloads are broadly distributed, we add one and apply a logarithmic transformation to reduce their skewness, i.e. we take $\log (C+1)$ and $\log (S+1)$. Table 3 in the “Appendix” section reports Pearson’s correlation coefficients between $\log (W)$ and each of the two performance measures. The negative and significant result confirm that those publications which are handled faster have indeed a higher performance both in terms of citations and downloads. This result is consistent with previous findings (Shen et al. 2015), which suggested that there is a negative correlation between citation counts and manuscript handling times.

These findings indicate that our observed correlation between editor–author distance and manuscript handling times could, in fact, be a confound of article performance (and thus quality). To disentangle the contribution of article performance from the editor–author distance, in the following we fit two extended log-linear regression models. These two models explain the handling time as a combination of editor–author distance and the two performance metrics individually. However, before defining these models, we need to test for correlations between editor–author distance and the two performance metrics. The reason for this is that, if such a correlation exists, we must account for it in our model. In Table 4 in the “Appendix” section we thus use a linear-regression model to assess the influence of $\delta _{D,1}$ on $\log (C+1)$ and $\log (S+1)$ respectively. The results reveal that there is a significant correlation between $\delta _{D,1}$ and *citations* but not between $\delta _{D,1}$ and *downloads*. This implies that we can assume downloads to be independent of $\delta _{D,1}$, while we need to account for the correlation between $\delta _{D,1}$ and $\log (C+1)$ in the model correcting for citations. Given these relationships, we define the two following *combined* models:

3 $$\begin{aligned} \log (W)&= \left[ \alpha ^s + \beta ^s \log (S+1)\right] + \delta _{D,1} \left[ \alpha ^s_{1}\right] \end{aligned}$$

4 $$\begin{aligned} \log (W)&= \left[ \alpha ^c + \beta ^c \log (C+1)\right] + \delta _{D,1} \left[ \alpha ^c_{1} + \beta ^c_{1}\log (C+1)\right] \end{aligned}$$

Equation (3) models the handling time as a linear combination of distance $\delta _{D,1}$ and downloads $\log (S+1)$. We refer to this model as Model 2. Its coefficients $\alpha ^s$ and $\alpha ^s_1$ play the same role as in Eq. (2), capturing the baseline manuscript handling time and the additional linear effect for publications with editor–author distance $D=1$. The coefficient $\beta ^{s}$ accounts for the additional effect of downloads $\log (S+1)$ on manuscript handling times. If fitting the model yields a significant negative value for $\alpha ^s_1$, this indicates a decreased handling time that cannot be explained by the article quality, as proxied by the number of downloads.

Equation (4) models the handling time as a linear combination of distance $\delta _{D,1}$, citations $\log (C+1)$, as well as their interaction (thus accounting for the correlation between $\delta _{D,1}$ and $\log (C+1)$ identified above).^4^ We refer to this model as Model 3. In addition to $\alpha ^{c}$ and $\alpha _1^{c}$ with interpretations analogous to above, it includes a coefficient $\beta ^{c}$ accounting for the effect that citations have on the manuscript handling times of *all* publications. Moreover, we include an interaction coefficient $\beta _{1}^{c}$, which captures the *additional* effect that citations have on the handling time of those manuscripts with editor–author distance $D=1$.

Table 1 reports the fitted coefficients of the Models 2 and 3. First, we find that the estimate of $\alpha _{1}$ is negative and significant for all models (i.e. Model 1, 2 and 3). As such, all of the regression models confirm that manuscripts submitted by previous co-authors of the handling editor are, on average, accepted faster. Moreover, the fitted coefficients for Models 2 and 3 confirm that this influence of the editor-distance also holds if we control for the performance of the publication. As can be seen in Table 1, this effect even becomes stronger if performance measures are additionally taken into account. A high number of citations and downloads shortens the handling time of all publications, as shown by the negative estimates of the coefficients $\beta ^c$ and $\beta ^s$. Notably, the only coefficient that is not significant is $\beta _{1}^{c}$. This indicates that, for those manuscripts submitted by previous co-authors of the handling editor, we cannot conclude a difference in the magnitude of the effect of quality in manuscript handling time.

**Fig. 4:**
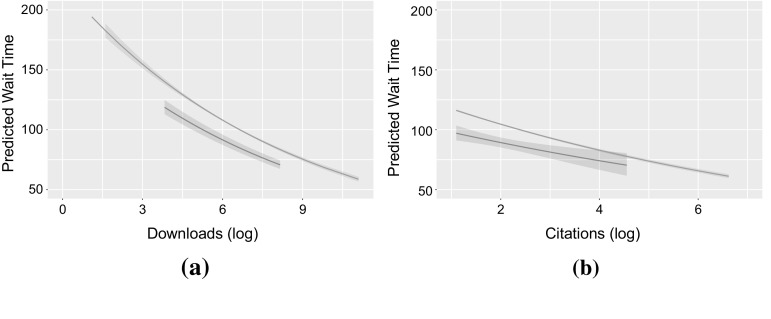
Predicted manuscript handling times for $D=1$ (*blue*) and $D>1$ (*red*). *Shaded areas* show 95% confidence intervals of the prediction, **a** manuscript handling times predicted by the model depending on the number of article downloads, **b** manuscript handling times predicted by the model depending on the number of citations. (Color figure online)

**Fig. 5:**
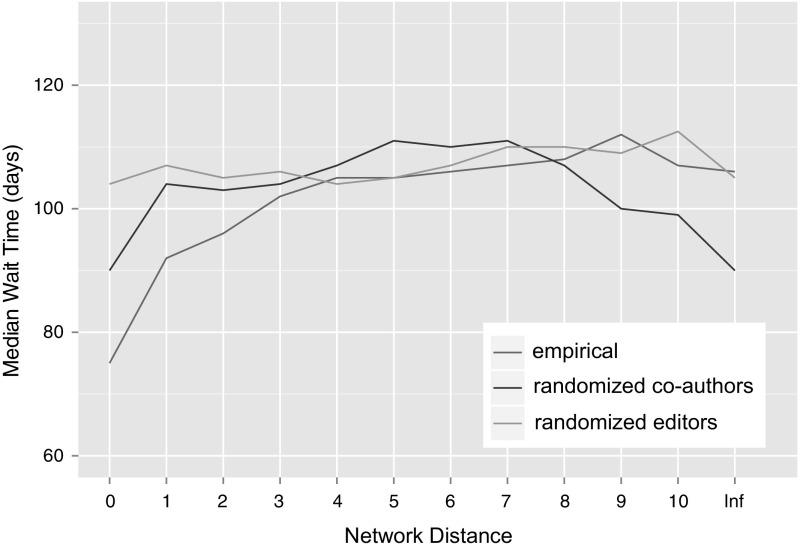
Mean time to acceptance of articles at various network distances for the empirical network and the two randomized networks. Shorter acceptance times can be observed at distances one and two, becoming very close at distance three and beyond

**Fig. 6:**
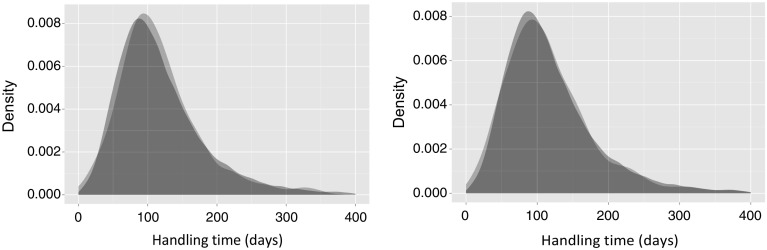
Kernel density plots $({\hbox {bandwidth}} = 0.8)$ of the conditional distributions of *W* given $D=1$ (*red*) and $D>1$ (*blue*) in 50 simulations of the randomized editor network (*left*) and of the randomized co-authorship network (*right*). There is no clear shift between the conditional distributions for the two shuffled networks. (Color figure online)

We complement our study of how performance measures and editor distances influence manuscript handling times by taking a different perspective on the fitted parameters of Model 3. For this, we use the fitted model coefficients for the two cases $\delta _{D,1}=0$ and $\delta _{D,1}=1$ separately, and visualize how the manuscript handling time *W* predicted by the model depends on (1) the number of downloads *S* and (2) the number of citations *C* of an article. The predicted handling times are shown in Fig. 4a, b.^5^

This visualization of the fitted coefficients highlights several interesting findings of our analysis: first, both in Fig. 4a, b), we observe that both curves exhibit a negative slope, which corresponds to the significantly negative estimate of the model coefficients $\beta ^s$ and $\beta ^c$. An intuitive interpretation of this striking finding is that manuscripts which are handled faster by editors are indeed likely to have higher *quality* (as indicated by the future number of downloads and citations). Secondly, both for the number of downloads in Fig. 4a and the number of citations in Fig. 4b, we observe a clear shift in the vertical direction between (1) the predicted handling time for articles with $D=1$ (shown in blue) and $D>1$ (shown in red). This observation corresponds to the negative estimates of the model coefficients $\alpha ^S_1$ and $\alpha ^C_1$. Figure 4 shows that this shift holds *throughout the whole range* of article performance metrics, both in terms of downloads and citations. A natural interpretation of this finding is that—independent of the *quality* of a manuscript—those manuscripts with an editor–author distance of one are *always* handled faster than those manuscript where the distance is larger. Finally the fitted interaction coefficient $\beta ^c_1$ appears as a soft difference between the intensity of these trends in the citations case. Moreover, a small narrowing can be observed for those articles with a large number of citations. This finding suggests that the effect of social relations, as captured in terms of the author–editor distance, on manuscript handling times is less pronounced for very highly cited articles (but still present).

**Fig. 7:**
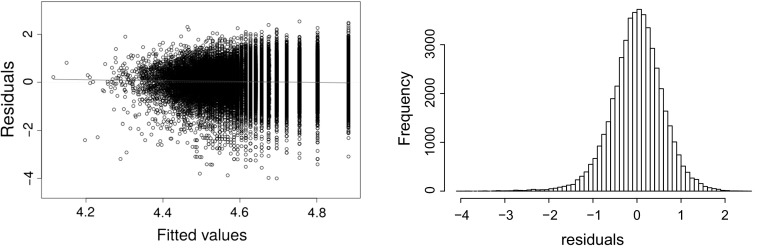
Residuals versus fitted values (*right*) and the histogram of residuals (*left*) for the model of handling time controlling for citations

**Fig. 8:**
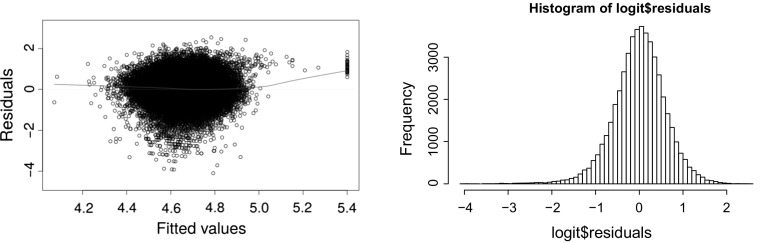
Residuals versus fitted values (*right*) and the histogram of residuals (*left*) for the model of handling time controlling for downloads

To verify the adequacy of our regression models, we have performed a diagnostic analysis and the results of this analysis are shown in Figs. 7 and 8 in the “Appendix” section. We find that the residuals of both fits are approximately normal and unbiased, which shows that assumptions underlying the log-linear regression model hold. In order to control for potential temporal inhomogeneities in the observed statistical dependencies, we further stratified our analysis by repeatedly fitting our regression model for co-authorship networks filtered by a given year. As shown in Table 5 in the “Appendix” section, all of the statistically significant fitted coefficients are qualitatively similar to those for the whole corpus. Furthermore, we tested the role of time between publication date and the date of the collaboration between handling editors and authors. We fitted a model with dummy variables for various values of this lag, finding that the coefficient of $D=1$ is negative and significant across values of the lag, as reported in the “Role of time between collaboration with editor and publication” section. This shows that our conclusion that the handling time of manuscripts with editor–author distance of one is significantly shorter than that of other manuscripts (1) does not sensitively depend on the chosen time frame, and (2) is statistically significant for data on each year after 2010 individually.

We tested the possible role of confounding factors in our results in an additional regression model taking into account various control variables, including editor experience, topic similarity, and amount of coauthors. We found that the effect of $D=1$ in handling times is robust against the inclusion of these controls (see “Correcting for experience, connectedness and topical similarity” section), highlighting the robustness of our findings. Finally, we repeated the same analysis but testing if citing the handling editor in the publication is also related to shorter handling time. We found no significant effect, as reported in the “Correcting for experience, connectedness and topical similarity” section, illustrating an alternative condition in which some relation to the editor is not related to different handling times.

### Reciprocity between editors

So far we have considered relationships between editors and authors which are based on co-authorship relations. However, we can think of other social relationships which are not based on coauthorships. As an example, editors could have reciprocal relationships in which one editor handles publications authored by the other and vice versa. Such reciprocal relationships can occur even if these editors have not previously coauthored an article.

Our data set allows us to test whether such reciprocal relations exist and to what extent they impact handling times of publications. Formally, we define a reciprocal editor relationship between two editors ${\hbox {e}}^a$ and ${\hbox {e}}^b$ if there are two publications $p^{i}$ and $p^{j}$ such that ${\hbox {e}}^a$ is author of $p^i$ and editor of $p^j$, and ${\hbox {e}}^b$ is author of $p^j$ and editor of $p^i$. Since each reciprocal relationship is necessarily based on two publications $p^i$ and $p^j$, we can identify the younger of these two publications, i.e. the one which was submitted later. We call this younger publication (e.g. $p^j$) the *reciprocating publication*, while we call the older one the *preceding publication* (e.g. $p^i$). Notably, reciprocating publications are necessarily handled by an editor whose own (preceding) publication was previously accepted by one of the authors of this publication.

In our data set, we identify 330 reciprocating publications. In the following, we measure the handling time of *reciprocating publications* as $W^j$ and compare them to the handling times of (1) all other publications, (2) publications that have been authored by an editor but were not reciprocating, and (3) the corresponding *preceding publication*, with handling time denoted as $W^i$. We first test the hypothesis that the handling times $W^j$ of *reciprocating publication* are significantly smaller than those of all other publications. A Wilcoxon test shows a significant difference of 28 days between the medians of both distributions (confidence interval [23, 34], $p<10^{-15}$), i.e. reciprocating publications are on average accepted 4 weeks earlier than other manuscripts.

This difference can either be attributed to the reciprocal editor relation, or to the fact that editors, in general, write articles that are accepted faster. To discern between these two alternative explanations, we compare $W^j$ with the handling times $W^{{\mathrm{ed}}}$ of all other manuscripts who have an author that is also an editor. A Wilcoxon test reveals a difference of 24 days between the medians of both distributions (confidence interval [18, 30], $p<10^{-14}$), i.e. reciprocal publications are, on average, accepted more than 3 weeks faster than other articles co-authored by an editor of PlosOne. The result of this second test supports the hypothesis that it is indeed the reciprocal relationship between editors that is responsible for the reduction in handling times, rather than the editors experience or reputation.

We finally analyze the relationship between the handling times $W^j$ of reciprocating publications and the handling times $W^i$ of their corresponding preceding publications. The Spearman correlation coefficient between $W^j$ and $W^i$ is 0.40 ($p<10^{-12}$), suggesting that a faster handling of the preceding publication is related to a faster handling of reciprocating publication. To quantify this dependence, we fit a linear regression model in which $\log (W^j)$ is expressed as a linear function of $\log (W^j)$, shown in Table 2. These results give a clear message: the handling time of reciprocating articles is shorter when the handling time of the preceding publication was shorter. A possible explanation for the above results is the hypothetical existence of quid pro quo relationships between editors, in which an editor can get faster future treatment as author when they handle faster the previous papers submitted by another editor. While that constitutes an explanation, more direct evidence is necessary to conclude whether such exchanges of influence exist.

**Table 2:** Regression results for the model $\log (W_{j})= a + b \times {\hbox {log}}(W_{i})$

	$$\log (W_{j})$$
$\log (W_{i})$ (b)	0.43934***
Constant (a)	2.38039***
*N*	330
$R^{2}$	0.076597
Adjusted $R^{2}$	0.1731

* $$p< 0.05$$; ** $$p < 0.01$$; *** $$p< 0.001$$

## Discussion and conclusions

Just like any other human endeavour, the academic peer review process is not free of prejudices and social biases (Merton 1968). While this problem is mostly discussed with respect to the reviewers of a publication, in our paper we focus particularly on the role of *handling editors* and their relation to the authors of the manuscripts they handle.

Our view is motivated by the fact that the handling editor (1) already controls whether a submission is subject for peer review or gets desk rejected, (2) selects the reviewers and thus potentially creates a bias toward acceptance or rejection, and (3) interprets the reports of reviewers to identify conflicts between reviewers and authors or problems with the reviewers. Constituting an admittedly extreme example the challenges that handling editors face, a number of scientists have recently been found to use forged online identities of scientists to provide favorable reviews of their own submissions (Ferguson et al. 2014). It is the task of the handling editor to detect, with the technical support of the publisher, such cases of blatant misconduct and to draw conclusions.

Acknowledging the key role of the handling editor in the review process, we want to quantify to what extent her performance, measured by the time it takes to handle a manuscript, is influenced by social and quality factors. This bears some limitations, which are partly due to the available data set from PlosOne. First of all, we have only data about manuscripts *accepted* for publication, not about rejected or withdrawn manuscripts. Hence, the handling time *W* always refers to the time span from initial *submission* to final *acceptance*. Secondly, our main variable *D*, the network distance between submitting authors and handling editor in a co-authorship network, is calculated on a co-authorship network constructed from publications in PlosOne. We are particularly interested in the case $D=1$, i.e. the handling editor is a previous co-author of one of the authors of the submission she is handling. But for our analysis, we have no information available about potential co-authorship *outside* PlosOne. Hence, there can be many more cases of previous co-authorship between authors and handling editor than the 1067 publications detected in the PlosOne data set. Integrating coauthorship data across journals requires advanced name disambiguation methods and the integration of multiple heterogeneous data sources. This is a challenge worth investigating in future research and it follows from our results about the subset of PlosOne collaborations.

Thirdly, the social and quality factors have to be *proxied* by measures derived from the available data. For example, the *social relations* between authors and handling editor are proxied by the network distance *D* calculated on the co-authorship network. This leaves out other potential sources of social influence, such as having the same affiliation, membership in the same scientific board, collaboration in research projects, etc. While consistently analyzing all possible sorts of scientific influence requires a multiplex network approach combining links from various data sources, we have investigated some additional social relations (citations, editor reciprocity) discussed below. The *quality* of a submission is proxied by two variables that refer to the *performance* of the manuscript *after publication*. Our assumption is that the handling editor is able to estimate this quality when handling the manuscript, which is then confirmed by the later success of the publication. Article performance is proxied by the number of downloads and the number of citations of the respective publication, which of course are rather crude proxies of quality.

With these considerations in mind, we now summarize our findings, to put them into perspective regarding publication practice afterwards.

1. The case that editors handle a submission of previous co-authors ($D=1$) occurs more than twenty times more often than expected at random. Even the case that the handling editor and the submitting authors have a common co-author in another publication ($D=2$) occurs more than three times more often than at random. This finding points to a rather strong social relation coming from previous collaborations. It should be less surprising when keeping in mind that authors and handling editors often belong to the same scientific community. Still, it bears potential conflicts of interest when handling the submissions of close collaborators.
2. Editors handle the submissions of previous co-authors ($D=1$) significantly faster, with a reduction of 19 days on average, as compared to the rest ($D>1$). The reduced handling time for previous co-authors is a robust finding, even if controlled controlling for other factors, such as the quality of the submission, the experience of the editor, and the topical similarity. This means that the shorter handling time of the submissions of previous co-authors cannot be explained by the fact these submissions are of better quality or more related to the expertise of the handling editor. Other possible causation mechanisms can generate this pattern, for example editors desk-rejecting low quality papers of their previous collaborators or authors sending better work to editors that were their collaborators earlier. It is left open to formulate a theory that integrates these mechanisms and design studies that can differentiate them under the appropriate conditions.
3. Independent of the handling editor, we find that the handling time decreases with increasing quality of submissions. This confirms previous findings of Shen et al. (2015) and Lin et al. (2016). If we were to only attribute this effect to the handling editor, it implies that the editor is able to judge the better quality of the submission and, thus, to handle it more efficiently. But also reviewers may contribute to this effect, being able to write their report faster and more easily if the manuscript is of higher quality.

These main findings are complemented by a number of interesting observations:

1. *Effects at longer distances*: we find indications that shorter handling times might still exist at moderate distances longer than one, i.e. editors at distance two in the coauthorship networks might still handle submissions faster. Measuring the reach of shorter handling times with network distance requires information on collaborations beyond a single journal, a question that remains open for future research.
2. *Difference between citations and downloads*: both variables are used in our investigation to proxy the quality of the submission. Regarding their overall impact on the main findings above, they behave similarly. However, only one of these, namely the *citations*, shows a correlation with the author–editor distance $D=1$. Citations measure the impact of a publication (and, consequently, of the journal), whereas downloads rather measure the *popularity*. Hence, in terms of citations, we find that articles with an author–editor distance $D=1$ have slightly higher scientific impact but not higher popularity.
3. *Editor citations*: as discussed in the “Introduction” section, there is a positive relation between editorship and the number of citations an editor receives as an author. Submitting authors might expect some advantage from citing the handling editor, for example as a shorter handling time. We investigated this possible relation in our data set by repeating our controlled regression using as explanatory variable whether the submission cites a previous work of the handling editor, rather the $D=1$ relationship. We found no significant effect, as shown in “Correcting for experience, connectedness and topical similarity” section, revealing that we do not have evidence that citing the handling editor has an effect on handling time.
4. *Reciprocity between editors*: handling times are shorter not only for submissions by the co-authors of the handling editor, but also for articles submitted by other editors who previously handled the articles of the handling editor. This result points to a possible relationship between editors that calls for a revision of the incentives and practices of editors across journals.

Eventually, we discuss how our main finding, the significant reduction of manuscript handling times for (a) previous co-authors/previous handling editors and (b) high quality submissions, relates to editor and journal performance. In the “Introduction” section, we listed four different dimensions for such performance, (1) impact, (2) revenue, (3) reputation, (4) popularity. As we confirmed in our study, the handling editor is able to detect quality submissions. This correlates positively with *impact*, as measured by the number of citations, and *popularity*, as measured by the number of downloads. Shorter handling times also positively influence *reputation*, as long as they indicate a fast and reliable manuscript handling.

There is, however, a potential risk to reputation as previous co-authors and previous handling editors might be seen as receiving preferential treatment and thus general reputation can decrease. It is left to evaluate the impact of all these effects on the *revenue* of the journal, testing whether the journal could economically benefit from an incentive scheme in which only some submissions are processed faster.

The results of this study remind us that *editors are humans*, and as such they are subject to introduce a social bias in the functioning of the scientific community at large. Combining the transparent and open policy of PlosOne with large data processing techniques, we have been able to detect and diagnose the existence of relationships between the handling time of articles and author–editor relations. Our approach thus offers a mechanism for journals and regulators to monitor such undesirable differences, motivating future data-driven editorial policies that can ensure a fair, transparent, and unbiased handling of submissions.

## Appendix

### Handling times in randomized networks

We measure median handling times in the empirical data and simulations for articles with longer network distances. Figure 5 shows an important difference in the median time between data and simulations when at distance one, with some difference at distance two, and almost no difference at distance three.

We compared our empirical finding of faster handling times for articles with $D=1$ against simulations of the *randomized editor* and *randomized coauthorship* networks. Figure 6 shows the distributions of handling times conditional on $D=1$ versus $D>1$ for both randomization schemes. There is no evident difference between both cases in both randomization schemes, showing that the empirical effect disappears in these null models.

### Analysis of performance measures

See Tables 3 and 4.

**Table 3:** Pearson correlation coefficients and Bonferroni corrected *p* values between ${\hbox {log}}(W)$ and performance measures

	Correlation	*p* value
$\log (C+1)$	−0.184	${<}10^{-15}$
$\log (D+1)$	−0.152	${<}10^{-15}$

**Table 4:** Regression results of performance measures as a function of $\delta _{D,1}$

	$\log (C+1)= a + b \times \delta _{D,1}$	$\log (S+1)= a + b \times \delta _{D,1}$
*a*	1.877***	6.152***
*b*	0.111**	0.050 $(p=0.102)$
*N*	48,482	48,482
$R^2_{{\mathrm{adj}}}$	0.0001513	0.000035

Articles that satisfy $D=1$ have slightly higher amount of citations but not of downloads

* $$p<0.05$$; ** $$p<0.01$$; *** $$p<0.001$$

### Diagnostics on model fits

Figure 7 outlines the adequacy of the model we used in explaining the relation between handling time, citations, and $\delta _{D,1}$. The left panel of the figure shows that residuals are largely unbiased with respect to the fitted value, and the panel on the right shows the histogram of residuals which are approximately normally distributed. Similarly, Fig. 8 outlines the adequacy of the model model controlling for downloads.

### Yearly stratified regression analysis

See Table 5.

**Table 5:** Regression results stratified per year for the model of $\log (W) = \alpha + \alpha _1 \delta _{D,1}$, starting from 2008

Year	$\alpha$	$\alpha _1$
2008–2009	4.4***	0.14
2009–2010	4.5***	−0.23****
2010–2011	4.7***	−0.14*
2011–2012	4.7***	−0.23***
2012–2013	4.7***	−0.14***
2013–2014	4.7***	−0.23***
2014–2015	4.7***	−0.15***

While the very early fit does not give a significant negative estimate of $\alpha _1$, the effect of $\delta _{D,1}$ is significant and negative since 2009

* $$p<0.05$$; ** $$p<0.01$$; *** $$p<0.001$$; **** $$p<0.1$$

### Role of time between collaboration with editor and publication

To test a possible effect of time passing between publication date and the date of the collaboration between the editor and one author of the article, we fitted a model with dummy variables for $D=1$ at various years of this lag:

5 $$\begin{aligned} \log (W) = \alpha + \sum _L \delta _{D,1,L} \alpha _{1,L} \end{aligned}$$

Here $\delta _{D,1,L}$ takes value 1 if the publication was at distance 1 with a lag of *L* years and 0 otherwise, $\alpha _{1,L}$ quantifies the effect of publications at distance 1 with lags of *L* years, and the values of *L* range from 0 (if published later the same year) to 4 years, with an additional value for lags of 5 years or more. The results, shown on Table 6, reveal that all values of $\alpha _{1,L}$ are negative and significant, highlighting that the shorter handling time of papers with $D=1$ is consistent across lags between publication dates and collaborations between submitting authors and handling editors.

**Table 6:** Regression results taking into account time lag between the most recent collaboration of the submitting authors with their editor and submission date

	log(w)
$\alpha _{1,0}$	−0.188718***
$\alpha _{1,1}$	−0.139132**
$\alpha _{1,2}$	−0.176558**
$\alpha _{1,3}$	−0.227453**
$\alpha _{1,4}$	−0.264907**
$\alpha _{1,5+}$	−0.280685*
$\alpha$	4.664338***
*N*	48,482
Log likelihood	−44,279.930000
AIC	88,573.870000

The effect of an article being at distance one on waiting time is significant for all time spans

* $$p < 0.05$$; ** $$p< 0.01$$; *** $$p< 0.001$$

### Correcting for experience, connectedness and topical similarity

Finally, apart from the factors outlined in the “Results” section, the handling time of manuscripts can be influenced by other factors such as (1) the experience of an editor, (2) the number of collaborators of an editor (which could serve as potential reviewers), (3) the topical similarity between the paper and the editor’s expertise, (4) the number of collaborators of authors, and (5) the amount of authors of the publication. In the following, we test whether our results are robust if we control for these additional parameters. For this, we quantify *editor experience* in terms of the number of submissions handled by the editor prior to the current assignment. The underlying assumption is that editors who handled a larger number of submissions are more experienced and thus able to find reliable reviewers in a shorter time. We quantify the *number of collaborators* by the editors’ degree in the co-authorship network, i.e. their number of different coauthors. Here, the assumption is that editors with a larger author degree, have a large network of trusted colleagues that will help them to quickly identify competent (and reliable) reviewers. Finally, we quantify the *topical similarity* between the manuscripts handled by editors and their own publications by studying their overlap in terms of reference lists. The underlying assumption is that manuscripts with high topical similarity to the editor’s publications can be handled in shorter time, since the editor is an expert him or herself. Finally, the fact that an editor handles the submission of previous co-authors can also be a mere combinatorial effect if some of the authors of the submission have a large number of co-authors. In the following, we correct for this by additionally studying the *number of collaborators of authors*, in particular by the *maximum degree* of any author in the co-authorship network. Finally, we control for the *amount of co-authors of the article*, i.e. the length of the author list. This is necessary to correct for possible size effects stemming from larger co-author groups, which are more likely to have collaborated before with the handling editor.

This leaves us with five factors, whose influence on the handling time of manuscripts we will test in the following. To test the robustness of our main finding, we again combine these factors with $\delta _{D,1}$ (see definition above), which indicates whether the editor and authors have previously co-authored an article. We do this in two ways: first, including the five factors as a linear combination that extends the basic model of Eq. 2, and second by fitting first a multinomial model of $\log (W)$ and then testing a linear relationship between $\delta _{D,1}$ and the residuals of that model. Tables 7 and 8 present the results of these two tests, showing that the estimate of $D=1$ is negative and significant even when controlling for all these possible confounds.

**Table 7:** Regression results including linear controls for the models with $D=1$ and with cited editor from the published manuscript

	$D=1$	Editor cited
$D=1$	−0.178056***	
Editor cited		−0.047102
Amount of co-authors	0.003532***	0.003235***
Editor experience	−0.002272***	−0.002256***
Amount of collaborators of editor	0.000014	−0.000045
Max. amount of collaborators of authors	0.0000004***	0.0000004***
Topical similarity with editor	−0.217794***	−0.226042***
Intercept	4.704207***	4.705701***
*N*	48,482	48,482
Log likelihood	−43,694.380000	−43,719.460000
AIC	87,402.770000	87,452.920000

The result at $D=1$ is robust to the inclusion of controls and the effect of citing the editor is not significant

* $$p < 0.05$$; ** $$p< 0.01$$; *** $$p< 0.001$$

**Table 8:** Regression results after residualizing to a multinomial model of the control variables

	$D=1$	Editor cited
$D=1$	−0.168680***	
Editor cited		−0.045963
Intercept	0.002168	0.000584
*N*	48,482	48,482
Log likelihood	−43,658.030000	−43,680.890000
AIC	87,320.060000	87,365.770000

The result of $D=1$ is robust to these controls and the effect of citing the editor stays non-significant

* $$p< 0.05$$; ** $$p < 0.01$$; *** $$p< 0.001$$

Another possible influence of the handling editor could come from the fact that previous publications of the handling editor (as an author) are *cited* in the submission she handles. Different from the *undirected* relation of a previous co-authorship between the authors and the handling editor, a citation of the editor’s work creates a *directed link* that expresses scientific impact. We quantify this kind of directed relationship as a binary variable $E_{{\mathrm{cit}}}$ that takes the value 1 if the article cites a previous work of the handling editor, and zero otherwise. We fit the same controlled models as above, replacing $\delta _{D,1}$ for $E_{{\mathrm{cit}}}$, reported in Tables 7 and 8. The non significant estimates of citing the editor reveal that we do not have sufficient evidence to conclude that citing the editor is related to shorter handling times.

## Footnotes

1 Note that our low frequency crawler is compliant with the Terms of Use, and copyright licenses stated at http://journals.plos.org/plosone/.

2 However, since all handling editors selected for our data set have necessarily co-authored a PlosOne article, this implies that ${\hbox {e}}^i$ has co-authored a paper *after* the submission of $p^i$.

3
http://journals.plos.org/plosone/s/competing-interests.

4 Note that, since there is no significant correlation between $\delta _{D,1}$ and $\log (S+1)$, in Eq. (3) we do not need to include such an interaction term.

5 Note that, while we use a *linear* regression model on logarithmic handling times, downloads and citations, in Fig. 4 the resulting *linear* functions are plotted on a log-linear scale for better visibility.
